# Proximal humerus fractures (PHFs): comparison of functional outcome 1 year after minimally invasive plate osteosynthesis (MIPO) versus open reduction internal fixation (ORIF)

**DOI:** 10.1007/s00068-021-01733-w

**Published:** 2021-07-03

**Authors:** Laura Buchmann, Esther M. M. van Lieshout, Miliaan Zeelenberg, Dennis den Hartog, Roman Pfeifer, Florin Allemann, Hans-Christoph Pape, Sascha Halvachizadeh

**Affiliations:** 1 Department of Trauma, University Hospital Zurich, Raemistrasse 100, 8091 Zurich, Switzerland; 2 Faculty of Medicine, University of Zurich, Raemistrasse 75, 8006 Zurich, Switzerland; 3 Trauma Research Unit, Department of Surgery, Erasmus MC, University Medical Center Rotterdam, Rotterdam, Netherlands

**Keywords:** MIPO, ORIF, PHILOS, Proximal humerus fracture, Soft tissue, Osteosynthesis

## Abstract

**Purpose:**

Osteosynthetic treatment strategies of PHFs include MIPO or ORIF techniques. The aim of this study was to compare the 1 year outcome following either technique in type B PHFs.

**Methods:**

This study was designed as a retrospective cohort study of patients treated at one academic Level 1 trauma center. Patients from 2009 to 2019 who required surgical treatment of a type B PHF were eligible to be included in this study. Patients with A- or C-type fractures or patients requiring arthroplasty were excluded. All patients were treated with Proximal Humerus Interlocking System (PHILOS) and stratified according the approach into Group MIPO or Group ORIF. Outcome measures include local complications that occurred during hospitalization, nonunion after 12 months, and range of motion after 1 year follow-up.

**Results:**

This study included 149 (75.3%) patients in Group ORIF, and 49 (24.7%) in Group MIPO. The fracture morphology and concomitant injuries were comparable amongst these groups. When compared with Group MIPO, Group ORIF had a 2.6 (95% CI 0.6–11.7) higher risk of suffering from local complications. The rate of postoperative nerve lesions was comparable (OR 0.9, 95% CI 0.1–9.7) as was the rate of soft tissue complications (OR 2.0, 95% CI 0.2–17.2). The risk for nonunion was 4.5 times higher (95% 1.1–19.5) in Group ORIF when compared with Group MIPO. Group MIPO had a higher chance of flexion above 90° (OR 8.2, 95% CI 2.5–27.7).

**Conclusion:**

This study provides indications that patients following surgical treatment of PHFs in MIPO technique might have favourable outcome. Large-scale and high-quality studies are warranted to confirm these results.

## Purpose

PHFs account for approximately 5% of all fractures in adult patients and are in the top three causes of osteoporotic fractures [1]. The increasing incidence of PHFs might be explained by an increased life expectancy and more active lifestyle [2, 3]. The treatment strategy depends on the fracture morphology, activity level of the patients, and the soft tissue condition [4]. Non- or minimally displaced fractures qualify for potential non-operative treatment strategies [5]. Patients suffering from displaced fractures might, however, benefit from a surgical treatment strategy to improve shoulder function [6]. In general, the major surgical treatment strategies for PHFs include osteosynthesis and arthroplasty [7]. The proximal humeral interlocking system (PHILOS) has been reported among the preferred surgical modality in fractures that might benefit from osteosynthesis [8, 9]. However, to improve functional outcome, complications based on unthoughtful management of the soft tissue status, including infection, delayed union, or avascular head necrosis should be reduced [10]. Among others, the MIPO technique has gained popularity as a potential surgical treatment strategy for PHFs [11]. Several studies reported beneficial outcomes following MIPO, including the lower rate of vascular damage, shorter duration of surgery and reduced postoperative pain [12–14].

There still is ongoing discussion on the benefits of MIPO when compared with ORIF in type B fractures. Therefore, the aim of this study was to test the following hypothesis: MIPO results in lower complications rates and improved functional outcome 1 year after surgical treatment of type B PHFs.

## Methods

This study was designed as a retrospective cohort study and adheres to the “strengthening the reporting of observational studies in epidemiology (STROBE) statement” [15] and was conducted at a European academic level 1 trauma centre.

### Participants

Patients who suffered a PHF that required surgical treatment were eligible to be included in this study. Only patients who suffered type B fractures following the AO/OTA fracture classification [16] who were treated with PHILOS were included in this study. Patients were stratified either to Group MIPO or Group ORIF. The treatment strategy based on the preference of the leading surgeon (FA). The senior surgeon (FA) was the leading surgeon in all cases.

All patients were strictly treated following the PHILOS guidelines provided by DePuy Synthes (Oberdorf, Switzerland). Group ORIF was treated following a delto-pectoral approach with open reduction and PHILOS.

The approach in Group MIPO included an anterolateral deltoid split proximally and a lateral approach to the humerus distally. The correct approach at the distal site was verified under the fluoroscope. Patients were followed-up 6 weeks, 3 months, 6 months and 12 months after the surgery as part of the routine clinical aftercare. Patients who did not complete the follow-up at our clinic, had secondary intervention at the humerus or suffered from multiple injuries of the arm were excluded from this analysis.

### Variables and definitions

Outcome variables include complications and range of motion (ROM). Complications include postoperative nerve injury that was stratified to sensory or senso-motoric damage, injuries to the rotator cuff, and soft tissue complications including superficial or deep infection. Complications that were documented within 30 days after surgery were included in this study. The range of motion was quantified 12 months following the surgical intervention. To approximate clinical functionality ROM was quantified during the routine clinical follow-up and verified by the leading surgeon (FA). Functionality includes flexion above 90° and the Apley scratch test, where patients were asked to touch their lumbar spine area during adduction, internal rotation, and retroversion in the shoulder joint. The radiation time during surgery was collected from the fluoroscopic device. All surgeries were performed with the same setting and the same device.

Radiologic union was assessed 12 months after surgery. A nonunion was defined when at least the fracture line was still visible without any signs of callus formation.

Patients’ comorbidities were summarised according to Charlson comorbidity index (CCI) [17]. Trauma energy was stratified to low, medium, and high according to the definition by Tscherne [18]. Displacement was defined following the Neer classification [19].

### Data sources and study size

Data were extracted from the electronic medical records and based on clinical measurements that were taken during routine clinical work. This study includes a maximal available dataset; therefore, a formal sample size calculation was not performed.

### Statistical analysis

Continuous variables are summarised as the mean and standard deviation (SD), and categorical as number (*n*) and percentage. Group comparison was performed depending on distribution with the Students *t* test, Mann–Whitney *U* test, or depending on variable structure with the chi-square test. The level of significance was set at a *p* value < 0.05. All statistical analyses were performed using the R system for statistical computing and graphics, version 4.0.4 (R Core Team, 2021).

## Results

### Participants and descriptive data

**Fig. 1:**
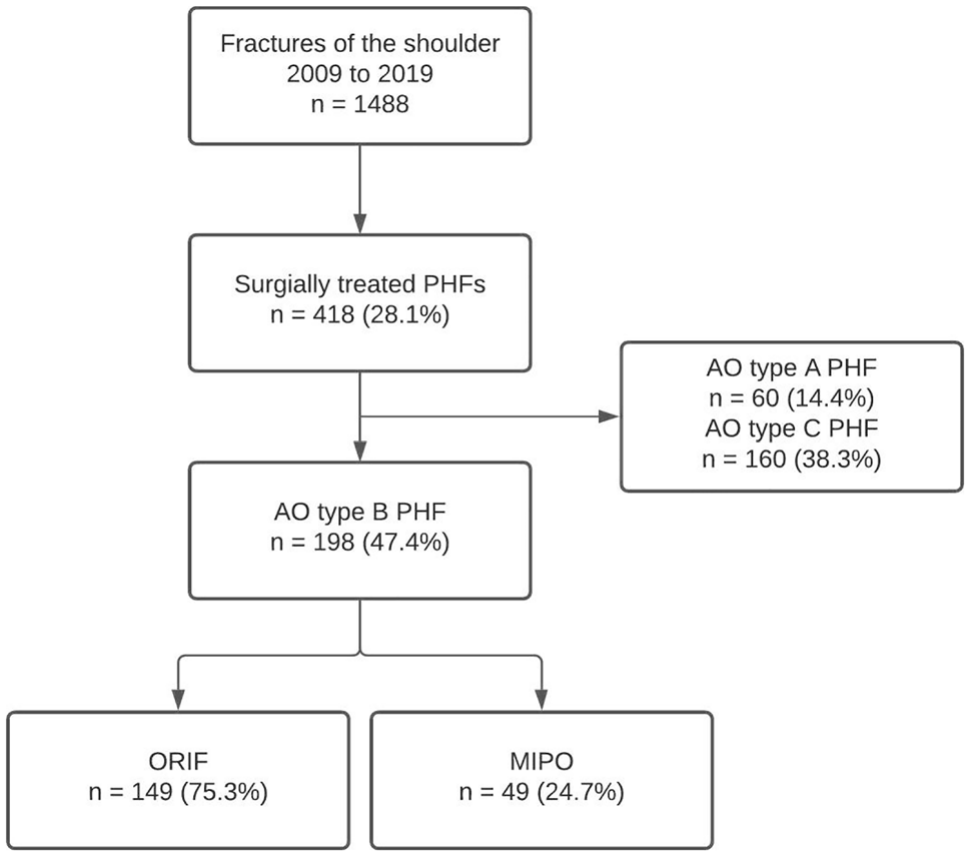
Flow diagram of included patients. *PHF* proximal humerus fracture, *AO* Arbeitsgemeinschaft für Osteosynthesefragen, *ORIF* open reduction internal fixation, *MIPO* minimal invasive plate osteosynthesis

During the 10 year period, 1488 patients were treated due to a fracture of the shoulder joint. Of these, 418 (28.1%) required surgical treatment of a PHF. In total, 198 (47.4%) fractures were classified as AO type B, 149 (75.3%) were stratified in Group ORIF, and 49 (24.7%) in Group MIPO (Fig. 1).

The mean age of the study population was 64.6 (SD 17.8) years and included 123 (62.1%) female patients. The mean CCI was 2.8 (SD 2.3) points ranging from 0 to 10 points. Most patients (*n* = 153, 77.3%) suffered a ground-level fall representing low-energy trauma mechanism and 21 (10.6%) patients suffered from an additional dislocation in the shoulder joint. The duration of surgery and radiation time where comparable in both groups (Table 1).

**Table 1:** Demographics of study population (Type B fractures)

	ORIF	MIPO	*p* value
*n*	149	49	
Age [years], mean (SD)	64.84 (17.95)	63.86 (17.84)	0.742
Female gender, *n* (%)	92 (62.2)	31 (63.3)	1
BMI [kg/m^2^], mean (SD)	26.86 (7.76)	24.62 (6.10)	0.257
CCI [points], mean (SD)	2.71 (2.10)	3.18 (2.34)	0.187
Trauma energy, *n* (%)			0.266
Low	111 (74.5)	42 (85.7)	
Medium	21 (14.1)	4 (8.2)	
High	17 (11.4)	3 (6.1)	
Dislocation, *n* (%)			0.316
None	131 (87.9)	46 (93.9)	
Subluxation	6 (4.0)	0 (0.0)	
Luxation	12 (8.1)	3 (6.1)	
Prim. Nerve injury, *n* (%)			0.432
None	144 (96.6)	49 (100)	
Sensory	3 (2.0)	0 (0.0)	
Motoric	2 (1.3)	0 (0.0)	
Duration of surgery [min], mean (SD)	121.33 (52.56)	108.02 (43.36)	0.141
Duration of radiation during surgery [s], mean (SD)	191.1 (165.4)	181.4 (131.6)	0.702

*n* number, *BMI*  body mass index, *CCI*  Charlson comorbidity index, *ORIF* open reduction internal fixation, *MIPO* minimal invasive plate osteosynthesis

#### Complications following surgical treatment of PHFs

One patient (2.0%) in Group MIPO reported persistent neuronal pain at rest as well as in motion for longer than 3 months, while another patient reported wound dehiscence. In Group ORIF, 15 (10.1%) patients reported a complication. Five (3.4%) patients reported transient postoperative nerve injury, three (8.7%) reported injuries of the axillary nerve, and two (1.3%) injuries of the radial nerve. In total, six (4.0%) reported soft tissue complication. The remaining four (2.6%) required reoperation based on screw perforation, and secondary humeral head necrosis. While not statistically significant, the rate of complications in Group ORIF was 2.6 (95% CI 0.6–11.7) times higher when compared with Group MIPO. In total, 28 patients (14.1%) had signs of radiological nonunion 12 months after surgery (2, 2.6% Group MIPO vs. 26, 10.6% Group ORIF, *p* = 0.049).

### Range of motion

The degree of internal and external rotation, adduction and extension were comparable amongst the groups at 12 months after surgery. Abduction was higher in Group MIPO (88.9°, SD 4.2° vs. 82.2°, SD 16.2°, *p* = 0.035) as was elevation (140.8°, SD 40.3° vs. 93.1°, SD 68.9°, *p* = 0.001). Patients in Group MIPO had a further higher documented range of flexion (139.1°, SD 30.2° vs. 115.0°, SD 42.2°, *p* = 0.005). Patients in Group MIPO had an 8.2 (95% CI 2.4–27.7) times higher change to be able to flex the shoulder joint above 90° at 12 months after surgery, when compared with Group ORIF. Similarly, Group MIPO had a 10.2 (95% CI 2.4–43.7) times higher chance of reaching the lumbar vertebral body 1 (L1) or higher during the Apley scratch test when compared with Group ORIF (Table 2).

**Table 2:** Odds ratio of outcome measures MIPO (ref.) vs. ORIF

	OR	95% CI	*p* value
Complications	2.6	0.6–11.7	0.252
Postop. Nerve lesion	0.9	0.1–9.7	1
Soft tissue complications	2	0.2–17.2	1
Radiologic nonunion 12 months after surgery	4.5	1.1–19.5	0.035
Flexion above 90°	8.2	2.4–27.7	< 0.001
Apley scratch test L1 and higher	10.2	2.4–43.7	< 0.001

*OR* odds ratio, *CI*  confidence interval

## Discussion

MIPO represents a safe and effective alternative to ORIF as a surgical treatment strategy for PHFs [20, 21]. The aim of this study was to compare complication rates and the range of motion in patients who had a surgical treatment of type B PHFs using either the MIPO or ORIF techniques. This study revealed the following points:

- The postoperative complication rate was comparable between the groups.
- The functional outcome 12 months after surgery was better in Group MIPO when compared with Group ORIF.
- ORIF had higher risk for nonunion 12 months after surgery.

The increase rate of nonunion in Group ORIF might base on the periosteal stripping and the soft tissue handling around the fracture [22, 23]. The rate of nonunion in the present study population is comparable with the literature [22, 23]. This supports the fact, that adequate soft tissue handling and cautious treatment of the periosteum might support bone healing.

No postoperative radial nerve injury was observed in Group MIPO, in contrast to two cases (1.3%) in Group ORIF. Iatrogenic radial nerve injury has been reported to range from 5.1 to 17.6% in ORIF of humerus shaft fractures [24, 25], which can also occur in MIPO techniques and during the treatment of PHF. Techniques for axillary nerve protections include visualization and tactile protection and have reduced the odds for iatrogenic axillary nerve damages substantially [26]. The rate of wound infection after ORIF is reported to range from 1.9 [27] to 3.8% [28] and 4% [4], which is comparable to the presented results. The rate of wound infection following MIPO was lower in the presented study (2.0%). It is well known that minimally invasive surgery is associated with a lower rate of soft tissue infection [29]. The rate of avascular necrosis is reported to range from 5.5 to 10% [30, 31], which is comparable to the presented results. Comparable to other reports [14], Group MIPO did not present with avascular necrosis of the humeral head, a complication that still can occur following MIPO [32].

Several studies investigated pain following MIPO or ORIF in PHFs. Some studies reported comparable VAS [33], while others documented less pain following MIPO [34]. The improved pain scores facilitates physiotherapeutic advancements and might, therefore, increase range of motion. Furthermore, the soft tissue preserving technique of MIPO favours bone healing and promotes improved functionality [35]. It has been reported that the rate of bone healing after MIPO is higher when compared with ORIF [23]. The minimal soft tissue disruption allows the earlier mobilisation of the shoulder, which further promotes healing and range of motion [36]. This might be based on the preservation of periosteal bridges, which could represent the last link of perfusion to the articular fragment [36]. This might further support the observation of lower rates of avascular humerus head necrosis following MIPO. The advantages of functional outcome, blood loss, and postoperative pain following MIPO have indicated beneficial outcomes, while the rate of complications remains comparable in selected cases [37].

### Limitations

This study presents with certain limitations. The sample size in the present study is comparable with studies investigating the effect of MIPO on proximal humerus fractures [38–40]. Compared to these studies, the present study population is selected very carefully and includes only type B PHFs in order to improve outcome comparability and reduce confounding. Second, one might argue that the measurements of range of motion are not-standardised and lack relevance. This study included range of motion as an outcome measure representing the most relevant functional motions. While functional scoring system might represent a wide variety of daily activities, they might mask certain limitations of movement. The outcome (radiation time, duration of surgery, complications, etc.) might be associated with the surgeon’s experience [41]. To minimize this bias, this study only includes cases, where the senior surgeon (FA) acted as the leading and supervising surgeon. The leading surgeon performed critical steps of the procedures (e.g. reduction), while other steps might have been performed by residents or fellows during their training.

### Conclusion

MIPO is a valid and safe alternative to ORIF in type B fractures. The complication rates are comparable; however, functional outcome and range of motion might be improved following MIPO. Further large-scale studies are warranted to investigate the benefits and limitations of these two treatment strategies.

## Data Availability

All data, materials, and codes are available upon reasonable request.
